# Callous traits in children with and without conduct problems predict reduced connectivity when viewing harm to others

**DOI:** 10.1038/srep20216

**Published:** 2016-02-02

**Authors:** Keith J. Yoder, Benjamin B. Lahey, Jean Decety

**Affiliations:** 1 The Child Neurosuite - Department of Psychology, University of Chicago, Chicago, Illinois; 2 Department of Psychiatry and Behavioral Neuroscience, University of Chicago Medicine, Chicago, Illinois; 3 Department of Public Health Sciences, University of Chicago, Chicago, IL.

## Abstract

The presence of elevated callous unemotional (CU) traits seems to designate a distinct group of children and adolescents with serious conduct problems. However, the extent to which CU traits impact the aversive reaction to harm is still a contentious issue. Here, we examined the effective connectivity seeded in the anterior insula and anterior cingulate cortex in a large number of children (N = 123, age 9–11, 60 females) with various levels of conduct disorder (CD) symptoms in response to visual stimuli depicting other people being physically injured. Perceiving others being harmed was associated with increased hemodynamic activity in the left amygdala and right temporoparietal junction (rTPJ). Children with higher callous traits showed less functional connectivity seeded in anterior cingulate with left amygdala and anterior insula. Conversely, CD symptoms were positively related to connectivity of insula with rTPJ. Overall, these results suggest that callousness is marked by the disruption of widespread cortical networks responsible for detecting and appropriately responding to important environmental cues, such as the distress of others.

Children who exhibit conduct problems (CP), such as aggression, cruelty, and theft, are more likely to behave antisocially and immorally as adults, are at a higher risk of developing psychopathy and present a serious public health challenge^1^,^2^. Moreover, scholars working to extend the specific personality traits which characterize adult psychopathy to younger populations have identified callous-unemotional (CU) traits as another important risk factor for serious conduct problems^3^,^4^. These affective and interpersonal aspects, such as the lack of guilt, remorse, and empathy, are associated with greater delinquency, both in children with and without CP, predictive of adult psychopathy, and are relatively stable across development^5^,^6^,^7^. Thus, identifying the impact of CU traits on neural processing during empathy-eliciting tasks in children could provide important insights into the neural mechanisms underlying the development of adult antisocial behaviors^8^.

Examining the neural response to the observation or imagination of others in distress or physical pain provides one of the most useful models of empathy^9^. Perceiving another individual in distress triggers an harm aversion response, which can be coupled with feelings of concern for that person, two basic elements of empathy^10^. Neuroscientific investigations, using functional MRI, magnetoencephalography, and electrophysiology have employed this paradigm in adult populations to identify associations between psychopathic traits and atypical neural activation and connectivity when viewing harm to others, both in forensic and nonforensic populations^10^,^11^,^12^,^13^,^14^. However, this work has only recently been extended to children with behavioral problems^15^,^16^,^17^,^18^,^19^, and it remains unclear to which extent CU traits influence functional connectivity during the perception of pain in preadolescents.

Early functional neuroimaging studies of empathy have utilized pain perception tasks, and demonstrated overlapping neural activation in anterior insula (aINS), dorsal anterior cingulate (dACC), brainstem, and cerebellum, both for the experience of somatic pain and the observation of another in pain or emotional distress^20^ (for a meta-analysis). While these regions are reliably recruited in studies of empathy for pain, greater activity in these regions during these tasks may not reflect empathic processing *per se*, but rather pain recognition^9^ and harm aversion^10^. The pain of others is an evolutionarily important cue that serves to capture an individual’s attention and, in healthy adults, motivate prosocial behavior^21^,^22^,^23^. Appropriately detecting and responding to such cues relies on the recruitment of a more general “salience network”, which integrates cognitive, affective, and physiological state information to appropriately respond to motivationally relevant events across the domains of nociception, negative affect, and cognitive control^24^,^25^. Within this neural network, the aINS and dACC have been argued to serve complementary input and output functions, respectively^26^. Thus, in the context of pain perception, aINS and dACC (sometimes called frontoinsula and anterior midcingulate cortices) coordinate widespread cortical and subcortical activity to determine the optimal response to internal and external events^24^,^25^,^27^.

Importantly, several studies of negative affect and harm processing in children have demonstrated associations between the hemodynamic activity within the aINS and dACC and both antisocial behavior and CU traits. In fact, a twin study proposed gray matter volume in dACC as an endophenotype for psychopathic traits^28^. One functional MRI study of boys aged 9–15 found that aggressive behavior scores were negatively correlated with neural activity in dACC when viewing negatively valenced images^29^. Another study with adolescents found that conduct disorder (CD) symptoms are associated with greater activity in insula, dACC, striatum, and amygdala for stimuli depicting accidental harm compared to neutral matched stimuli^16^. More recently, an fMRI study reported reduced activity in the insula and ACC, as well as superior frontal gyrus and amygdala to stimuli depicting physical harm in adolescents with CD or Oppositional Defiant Disorder (ODD) but only when they imagined the pain happening to another person^19^. Moreover, the interpersonal/affective dimension of the Psychopathy Checklist-Revised was negatively correlated with signal in the ACC. In a larger study of young males (age 10–16) using similar stimuli, those youth exhibiting conduct problems showed reduced activity in the aINS, ACC, and inferior frontal gyrus^18^. Importantly, CU traits were significantly related to decreased neural activity in the aINS and ACC, while CP symptoms were linked to greater activity in ACC.

Conduct problems and CU traits have also been shown to independently influence social cognition, though sometimes in opposing fashion. For instance, during affective compared to cognitive theory of mind (i.e. judging how someone will feel compared to judging what someone believes or wants), young males with conduct problems showed reduced activity in right amygdala and right aINS^30^. Moreover, as previously observed in ACC response during pain processing^18^, CU traits and CP symptoms exerted suppressor effects on right amygdala response, with higher CP symptoms independently associated with increased activity, but higher CU traits independently related to decreased activity.

Characterizing brain activity in terms of functionally segregated regions does not reveal anything about how different brain regions communicate with each other. Whereas standard contrast analyses create a ‘‘snapshot’’ of regional brain activity in response to a task or condition, functional connectivity analyses can identify patterns of communication between regions that contrast analyses may not detect^14^,^31^,^32^, and how callous unemotional traits might modulate such networks^33^.

To our knowledge, no study to date has examined how CU traits and CP symptoms impact the functional connectivity of the aINS and dACC (two critical nodes for empathy and harm aversion) when children view stimuli depicting other people being harmed. To fill this gap in the literature, we used functional MRI in a diverse sample of young children (age 9–11) while they engaged in a well-established pain perception paradigm with functional MRI^12^,^34^,^35^. Given their central role in saliency processing, dACC and aINS were chosen as seeds for a functional connectivity analysis. We hypothesized that children high in callous traits, irrespective of their conduct disorder symptoms, would be less sensitive to the pain of others, and thus, show reduced functional connectivity within the salience network. Additionally, this stimulus set includes both intentional and accidental harmful actions. This allowed us to examine the influence of intentionality on the neural encoding of harm. Children with high callous traits were expected to incorporate less of the intentionality information, and thus show reduced neural differentiation between intentional and accidental harmful actions in rTPJ, a region that plays a pivotal role in the extraction of intentionality^36^,^37^,^38^.

## Results

Following scanning, children rated the level of pain in a subset of scenarios as well as their own aversive response (e.g., how upset they felt). Across all participants, pain and aversiveness evaluations were positively correlated (*r* = 0.82, FDR *q* < 0.001). Influence of scenario type (Type), question type (Question), and Group (high risk vs. low risk) were assessed with repeated-measures ANOVA. There were main effects of scenario Type (F(1,72) = 272.17, FDR *q* < 0.001, *η*^2^ = 0.52) and Question type (F(1,72) = 92.60, FDR *q* < 0.001, *η*^2^ = 0.10), indicating higher ratings for Harm compared to No Harm scenarios (t(76) = 16.71, FDR *q* < 0.001) and higher ratings for the perceived pain than aversive response (t(76) = 9.71, *p* < 0.001). These effects were qualified by a Type x Question interaction (F(1,72) = 64.98, FDR *q* < 0.001, *η*^2^ = 0.06), indicating that the Harm-No Harm differences was greater for perceived pain than aversive response (t(76) = 8.20, FDR *q* < 0.001). After correcting for multiple comparisons, the Group x Type interaction became marginally significant (F(1,72) = 4.01, *p* = 0.098, *η*^2^ = 0.00), indicating a trend for Harm – No Harm differences to be greater in the low risk group than the high risk group (t(72.75) = −1.89, FDR *q* = 0.62). The relation between callousness and these ratings was assessed by correlating ICU-Callous scores against the Harm – No Harm average ratings. Callousness was significantly negatively correlated with aversiveness (*r* = −0.40, FDR *q* = 0.002), and showed a trend towards a similar effect for painfulness ratings (*r* = −0.26, FDR *q* = 0.051).

**Figure 1:**
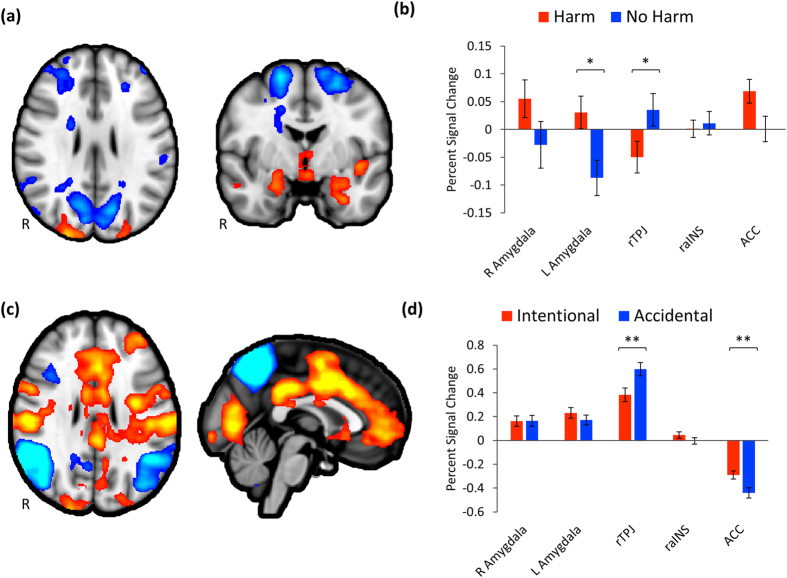
Group level results. Significant regions (corrected cluster *p* < 0.05) for the whole-brain contrast Harm – No Harm (**a**) and for *a priori* regions of interest (**b**). Harm – NoHarm became marginally significant in ACC *(q* = 0.071) after correcting for multiple comparisons. (**c**) Significant regions (corrected cluster *p* < 0.05) for the whole-brain contrast Intentional – Accidental and for *a priori* regions of interest (**d**). Error bars represent SEM. *FDR-corrected *p* < 0.05, **FDR-corrected *p* < 0.01.

**Figure 2:**
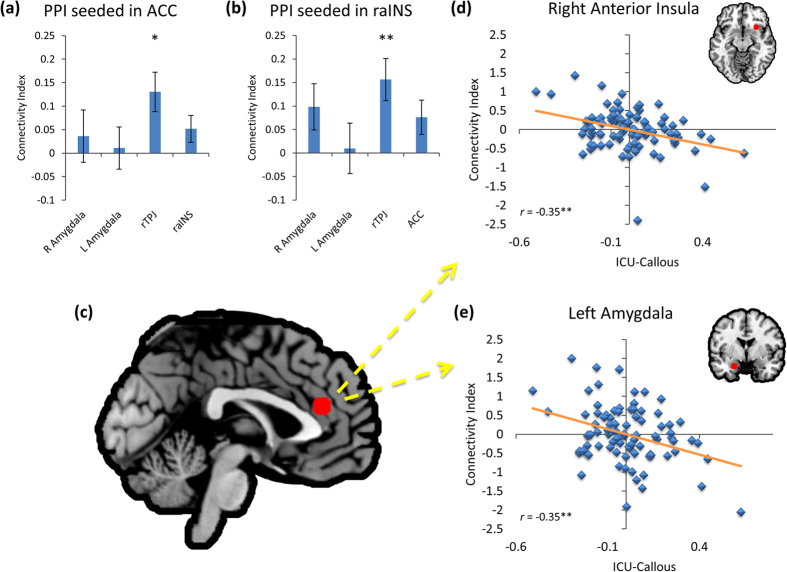
Region of interest analysis for psychophysiological interaction for Pain > No Pain. Percent change in connectivity seeded in ACC (**a**) or raINS (**b**) for ROIS. After controlling for ICU-Unemotional and ICU-Uncaring scores, as well as CD symptoms, age, gender, race-ethnicity, and maternal education, ICU-Callous scores predicted deceased functional connectivity seeded in anterior cingulate (**c**), with right anterior insula (**d**) and left amygdala (**e**). Plots show partial correlations, with the respective ROI marked in red. Error bars represent SEM. *FDR-corrected *p* < 0.05, **FDR-corrected *p* < 0.01.

Across all participants, there was significantly greater hemodynamic activity in the Harm condition within the left amygdala ROI (Fig. 1). The ACC ROI showed a trend towards the same effect (FDR *q* = 0.061). Conversely, rTPJ showed significantly greater activity during No Harm. Both ACC and raINS seeds demonstrated significant increased coupling with rTPJ/pSTS (Fig. 2). Activity within the ACC ROI was significantly greater for Intentional Harm, while rTPJ/pSTS response was greater for Accidental Harm (Fig. 1d). Activity in ACC was negatively related to aversiveness ratings (partial r = −0.32, FDR *q* = 0.017) and positively related to pain perception scores (partial r = 0.29, FDR *q* = 0.046). Postscan ratings were not significantly related to any other measure of hemodynamic activity or functional connectivity.

**Figure 3:**
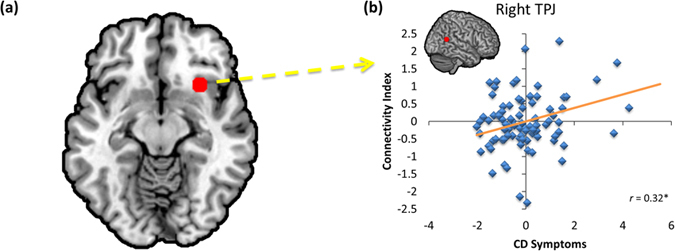
Region of interest analysis for psychophysiological interaction for Harm > No Harm seeded in right anterior insula. After controlling for ICU-Callous, ICU-Unemotional, and ICU-Uncaring scores, as well as age, gender, race-ethnicity, and maternal education, CD symptoms predicted increased functional connectivity between right anterior insula (**a**), and right TPJ/pSTS (**b**). Plot shows partial correlations, with the respective ROI marked in red. *FDR-corrected *p* < 0.05.

CU traits were assessed by parental report on the Inventory of Callous and Unemotional Traits (ICU), which includes separate subscales for callous, unemotional, and uncaring traits^39^. Standardized beta weights for each ICU subscores and CD symptoms (age, gender, race-ethnicity, and maternal education were also included as nuisance regressors) are reported in Table 1. After controlling for multiple comparisons, ICU-Callous scores were not significantly related to activity within any of the ROIs for the Harm *versus* No Harm contrast. ICU-Callous scores were negatively correlated with ACC-seeded connectivity with rTPJ and raINS (Fig. 2). For the PPI seeded in raINS, the rTPJ response was negatively related to callousness, but positively associated with number of CD symptoms. Symptom number was also related to significantly increased connectivity between raINS and right amygdala (Fig. 3). Follow-up analyses revealed no significant interactions with gender for either the whole-brain or PPIs.

## Discussion

The current study demonstrates the specific and independent influences of CU traits and CD symptoms on neuronal coupling when viewing others being harmed in a diverse sample of preadolescent children. As expected, when viewing harm to others, compared to visually similar control stimuli without harm, children showed greater activity in a host of regions, including bilateral amygdala, hippocampus, insula, MCC, and brainstem (Fig. 1). Similarly, the perception of harm was also associated with increased functional connectivity from ACC with rTPJ, as well as connectivity from raINS to rTPJ (Fig. 2). However, these patterns of connectivity were significantly impacted by children’s callous traits and, to a lesser extent, CD symptoms (Figs 2 and 3).

Neuronal activity in left amygdala was significantly greater for stimuli depicting harm (Fig. 1). Interestingly, we did not observe a significant negative relationship between callousness or CD symptoms and amygdala response (Table 1). This is a surprising finding since many studies investigating children with CD and callous unemotional traits^19^,^30^ (but see^12^,^16^), as well as studies of psychopathy in adults (see^40^ for a review) have reported an abnormal amygdala hemodynamic response to negative information. In our study CU traits are unrelated to amygdala response when witnessing harm to others. This may suggest that harm aversion is processed at an early stage in the amygdala, as documented by intra-cranial deep electrodes recordings^41^ as well as source localization with high-density EEG^42^ with the same stimuli, but, as indicated by the functional connectivity analysis, this information is not coupled with the dACC and aINS, which play pivotal roles in the interoception necessary for the construction of conscious affective experiences^43^.

For the PPI seeded in dACC, children with higher ICU-Callous scores showed reduced effective connectivity with right aINS and left amygdala (Fig. 2) when viewing depictions of others being harmed. Given their proposed function for detecting and responding to motivationally relevant information, especially in the context of negative associations between ICU-Callous scores and postscan ratings, the decreased connectivity between dACC and aINS suggests that individuals with high CU traits encode the pain of others as less salient than individuals with low CU traits. This fits with previous accounts of CU traits, as well as psychopathic traits^3^,^14^,^44^. Such an interpretation is also consistent with the observed reductions in left amygdala connectivity, since the amygdala plays an important role in motivation^45^.

The observed negative influence of callousness on amygdala-dACC connectivity is in line with models of psychopathy which posit amygdala disruption as a core feature^46^,^47^. A similar effect was recently reported between trait coldheartedness and amygdala-dACC coupling when healthy adults watched violence^14^. Thus, our findings suggest that childhood callousness and adult coldheartedness may both reflect specific disrupted communication between amygdala and dACC in the context of harm processing.

When right aINS was used as a functional seed, CD symptoms were related to increased neuronal coupling with TPJ/pSTS (Fig. 3). Similar stimuli have been used previously in incarcerated populations with mixed results. One study found that high levels of psychopathic traits were related to increased coupling seeded in right aINS with right TPJ/pSTS when participants imagined the action was happening to them^11^. In a different study, individuals with higher psychopathy scores demonstrated decreased functional connectivity seeded in rTPJ/pSTS with right aINS when viewing Harm compared to No Harm in a decision-making context^48^. Interestingly, previous of children with conduct problems have found suppressor effects between callousness and CP symptoms in the contexts of pain perception and cognitive and affective theory of mind^18^,^30^. Thus, future studies should continue to examine independent contributions of callousness and CP symptoms, and work to further distinguish between the two.

Unlike a previous study of callous traits in preteens^18^, at the whole-brain level, callousness scores were not significantly related to response in dACC or aINS. These differences could arise from the social nature of our stimuli (rather than simply hands and feet) or because we tested a slightly younger population (mean age of 10.5, compared to 13.7). However, an intriguing possibility is that the influence of callousness on neural functioning is first detectable in disrupted neuronal connectivity, and only later manifests as whole-brain differences in BOLD signal. Thus, future research will benefit from longitudinally measuring both neuronal responses as well as patterns of functional connectivity in order to better characterize how CU traits impact neural networks across development.

Overall, this study was designed to assess the influence of CU traits on neural function while children viewed others being harmed in a variety of social situations. After controlling for age, gender, CD symptoms, and maternal education, callousness scores uniquely predicted increased hemodynamic activity in right amygdala. Across all children, viewing harm was associated with increased effective connectivity between ACC and rTPJ, and between raINS and rTPJ. Moreover, callousness predicted reduced neuronal coupling seeded in dACC with left amygdala and right aINS.

Together, these data provide evidence in support of the hypothesis that children with high levels of callous traits do not respond appropriately to the harm of others because they experience a widespread disconnection in the cortical and subcortical networks which underlie important socio-emotional behaviors, such as empathy. Further investigation will be needed to better clarify the specific contexts in which CU traits are associated with network disruption, and whether or not children with high levels of CU traits might benefit from therapies designed to improve the function and integrative capacities of these networks.

## Methods

### Participants

Recruitment was designed to obtain a diverse sample of 123 children (10.55 ± 0.99 years; 60 females; 69 African Americans, collection stopped after obtaining 60 children of each gender). Children were excluded from the final analysis for missing data (*n* = 7) or excessive movement (*n* = 9), leaving a final sample of 106 children (see Table 2 for demographic information). Flyers advertising for well-behaved children were placed in pediatric well-visit waiting rooms, and flyers calling for children with behavioral problems were placed in outpatient child mental health clinics. Telephone screening interviews were used to recruit children into high or low risk for meeting DSM-IV diagnostic criteria for CD. The DISC Predictive Scale (DPS) for CD was administered to parents, then children. The DPS consists of 8 “stem questions” from the Diagnostic Interview Schedule for Children (DISC-IV) CD module^49^ and predicts a full diagnosis of CD with high specificity and sensitivity^50^. Inclusion in the high-risk stratum was based on parent alone endorsing 2 or more DPS items, child along endorsing 3 or more items, or the parent and child collectively endorsing 3 or more separate items. The low-risk stratum contained only children for whom neither parent nor child endorsed any CD items. Equal numbers of high risk and low risk girls and boys were recruited. Exclusion criteria included head trauma resulting in loss of consciousness exceeding 15 minutes and presence of pervasive developmental disorder. On the day of scanning, trained interviewers administered the full DISC-IV^49^, including the module for CD symptoms within the past 12 months, to the primary caregiver and the child in separate rooms. Parents also completed the Inventory of Callous and Unemotional Traits^39^. Parents provided written informed consent, and children gave informed assent. All study procedures were approved by the Institutional Review Board at the University of Chicago. All methods were performed in accordance with the approved guidelines.

### Stimuli and task

Prior to MRI scanning, participants were acclimated to study procedures in a mock scanner. This included lying in the mock scanner and practicing holding still while watching a documentary. Additionally, participants listened to recordings of scanner pulse sequences, and watched example stimuli from each condition (that were not used during the actual MRI scanning session).

Once in the magnet, participants viewed dynamic visual stimuli depicting either one or two individuals, with one person either being harmed (Harm), or not (No Harm). In the two person scenarios, harm was inflicted either intentionally or accidentally. Thus, there were five possible stimuli categories. Scenarios consisted of three pictures shown sequentially to suggest visual motion (1000 ms, 200 ms, 1000 ms). Participants viewed stimuli in alternating blocks of fixation cross (17.8 s duration) and task (20 s duration). The 20 task blocks were presented in pseudorandom order and consisted of six scenarios of the same type interspersed with a fixation cross (1134 ms). Stimuli presentation was controlled with E-Prime 1.0 (Psychology Software Tools, Inc., Pittsburgh, PA) and viewed via a back-projection system. Passive viewing was used to reduce cognitive load. Eye tracking was collected to ensure that children remained awake and attentive to the stimuli.

### Postscan ratings

After the scanning session, participants viewed 25% of the images again and answered questions using a visual analog scale (VAS). The VAS responses were coded so that they ranged from 0 to 100. Questions assessed harm perception (“How painful was it for the person who was hurt?”) and personal aversive response (“How upset are you when you watch this?”). Scores were averaged across each stimulus type, then across stimulus types to obtain average painfulness and distress ratings for Harm and No Harm images. Finally, change scores were calculated by subtracting ratings in the No Harm scenarios from ratings of Harm scenarios.

### Scanning parameters

Participants were scanned at the Brain Research Imaging Center at the University of Chicago using a Phillips 3T Achieva Quasar scanner. First, high-resolution T_1_-weighted structural scans were acquired using the 3D MP-RAGE sequence (repetition time = 2000 ms, echo time = 25 ms, flip angle = 77°, matrix = 64 × 64, field of view = 224 mm). Next, a single-shot EPI sequence was used to acquire functional images in 4 mm-thick transverse slices oriented to the AC-PC line (skip gap = 0.5 mm, repetition time = 2000 ms, echo time = 25 ms, flip angle = 77°, matrix = 64 × 64, field of view = 224 mm, in-plane resolution = 3.5 mm × 3.5 mm).

### Image processing and analysis

MRI data were processed with the FMRIB Software Library (FSL) version 5^51^. After removal of skull and other non-brain voxels, EPI images were realigned with MCFLIRT, high-pass filtered, and smoothed (6 mm FWHM). For normalization, functional images were first registered to each individual participant’s structural scan with boundary-based registration^52^, then registered to the standard MNI152 template via linear transformation with 12 degrees of freedom.

General linear modeling was used for statistical analysis as implemented in FEAT. Because the goal of this study was to examine neural processing associated with the perception of harm across a range of situations, stimuli were collapsed into Harm and No Harm categories. These two broad categories were modeled separately in the GLM, beginning at the onset of the first picture in each block, and continuing until the end of the last picture of the block. Motion parameters were entered as nuisance regressors, and volumes with abnormally large motion artifacts were de-weighted in the design matrix. All participants had fewer than 16% percent of volumes with motion contamination (average 36.6 ± 12.6 out of 450 volumes).

At the second level, a second GLM was used, with ICU subscores, CD symptoms, age, gender, race-ethnicity, and maternal education entered as covariates. Completion of high school was used to assess maternal education, because it is reliably associated with tested child intelligence^53^,^54^,^55^. Number of CD symptoms was used, rather than a dichotomous classification scheme, because dimensional analyses are better suited for hypothesis testing^56^,^57^ and more biologically valid for mental disorders^56^. Using FSL defaults, clusters at the whole-brain level were defined with a z threshold of 2.3 then corrected according to Gaussian Random Field theory for a cluster threshold of *p* < 0.05.

Based on previous studies of socioemotional processing, some using the same stimuli, including a recent meta-analysis of morality, empathy, and theory of mind^35^,^58^,^59^, regions of interest were chosen for the right TPJ/posterior superior temporal sulcus (TPJ/pSTS; x = 62, y = −54, z = 16), ACC (x = 0, y = 36, z = 20), raINS (x = 30, y = 18, z = −12), left amygdala (x = −22, y = −2, z = −24), and right amygdala (x = 16, y = −4, z = −24). For each ROI, masks were generated by placing a sphere with radius 6-mm centered at each coordinate. The TPJ/pSTS ROI, taken from a “moral cognition” meta-analysis^59^, was chosen because this ROI’s hemodynamic response and effective connectivity have been previously shown to be responsive to harmful scenarios in healthy adults^60^, and are influenced by levels of psychopathic personality traits in inmates, particularly when passively viewing harmful scenes^48^. The ACC and raINS ROIs we selected both for their role as core nodes of the salience network and previous work demonstrating their causal influence on network activity during socioemotional processing^58^. Finally, bilateral amygdala ROIs were selected because the amygdala plays a critical role in harm perception in childhood^35^ and is reliably linked to variation in callous traits in adolescent males^30^ and psychopathic personality traits in adult inmates^11^,^12^.

Functional connectivity was assessed using a psychophysiological interaction (PPI). For the ACC and raINS seeds, mean activity within the ROI was extracted and used as the physiological regressor (Harm-NoHarm served as the psychological regressor). Again, at the second level, age, gender, race-ethnicity, and maternal education were entered as covariates of no interest. Previous work suggests that CU traits and CD symptoms often demonstrate suppressor effects^18^,^30^, so we sought to identify the independent influences of CU traits and CD symptoms with multiple regression analyses for each ROI. In addition to ICU scores and CD symptoms, age, gender, race-ethnicity, and maternal education were entered as nuisance regressors. Participants who demonstrated signal change or connectivity values more than three standard deviations away from the full sample mean in any analysis were removed as outliers (*n* = 14). Finally, the graphically sharpened method for False Discovery Rate (FDR) was used to correct for multiple comparisons^61^. Across all analyses, two-sided tests were used and significance was set at α = 0.05.

## Additional Information

**How to cite this article**: Yoder, K. J. *et al*. Callous traits in children with and without conduct problems predict reduced connectivity when viewing harm to others. *Sci. Rep.* **6**, 20216; doi: 10.1038/srep20216 (2016).

## Supplementary Material

Supplementary Information

## Figures and Tables

**Table 1:** Beta weights for influence of ICU subscores and CD Symptoms on regions of interest.

**Analysis**	**ROI**	**ICU-Callous**	**ICU-Uncaring**	**ICU-Unemotional**	**CD Symptoms**
Harm-No Harm					
	rTPJ	0.14	−0.12	−0.08	0.15
	R Amygdala	−0.15	−0.01	0.00	0.10
	L Amygdala	0.08	0.02	0.05	−0.17
	ACC	−0.07	0.14	0.16	−0.13
	raINS	−0.00	0.41	0.13	−0.06
PPI-ACC					
	rTPJ	−0.26*	−0.47*	−0.21	0.06
	R Amygdala	−0.25	−0.28	−0.06	−0.10
	L Amygdala	−0.47**	−0.26	−0.06	0.18
	raINS	−0.46**	−0.39	0.02	0.20
PPI-raINS					
	rTPJ	−0.18	0.24	0.09	0.39*
	R Amygdala	−0.10	0.11	0.21	0.29
	L Amygdala	0.01	0.15	0.06	0.14
	ACC	−0.19	−0.07	−0.05	0.16

*Note.* Models also included age, gender, race-ethnicity, and maternal education as nuisance regressors.

Abbreviations: ROI, region of interest; ICU, Inventory of Callous-Unemotional Traits; CD, conduct disorder; rTPJ, right temporoparietal junction; ACC, anterior cingulate cortex; raINS, anterior insula.

^*^FDR-corrected *p* < 0.05

^**^FDR-corrected *p* < 0.01;

**Table 2:** Participant characteristics.

	**Full sample (n = 106)**	**Low Risk (n = 53)**	**High Risk (n = 53)**	***P*** **value**^a^
Age (years)	10.5 ± 0.86	10.6 ± 0.93	10.5 ± 0.80	0.234
Females	50 (47%)	26 (45%)	24 (49%)	0.846
African Americans	59 (56%)	29 (49%)	30 (57%)	0.999
Mother completed high school^b^	87 (82%)	47 (89%)	40 (75%)	0.127
CD Symptoms	1.37 ± 1.89	0 ± 0	2.74 ± 1.84	<0.001
ICU-Callous	6.75 ± 4.16	4.74 ± 2.07	8.75 ± 4.74	<0.001

Except for percentages, values represent means and standard deviations.

^a^p values calculated using Welch’s two-sample t-test except for gender, race, and maternal education, where Fisher’s exact test was used.

^b^values represent percent completed high school.
